# Placebo-Controlled Discontinuation of Long-Term Acid-Suppressant Therapy: A Randomised Trial in General Practice

**DOI:** 10.1155/2015/175436

**Published:** 2015-07-12

**Authors:** Jon Eik Zwisler, Dorte Ejg Jarbøl, Annmarie Touborg Lassen, Jakob Kragstrup, Niels Thorsgaard, Ove B. Schaffalitzky de Muckadell

**Affiliations:** ^1^Research Unit of General Practice, Department of Public Health, University of Southern Denmark, J. B. Winslows Vej 9A, Odense, Denmark; ^2^Department of Emergency Medicine, Odense University Hospital, 5000 Odense, Denmark; ^3^Research Unit for General Practice and Section of General Practice, Department of Public Health, Copenhagen University, 1014 Copenhagen K, Denmark; ^4^Department of Internal Medicine, Regional Hospital Herning, 7400 Herning, Denmark; ^5^Department of Medical Gastroenterology, Odense University Hospital, 5000 Odense, Denmark

## Abstract

*Objective*. To investigate whether patients on long-term antisecretory medication need to continue treatment to control symptoms. *Methods*. A double-blinded randomised placebo-controlled trial in general practices in Denmark. Patients aged 18–90 who were treated with antisecretory drugs on a long-term basis were randomized to esomeprazole 40 mg or identical placebo. Outcome measures were time to discontinuation with trial medication due to failed symptom control analysed as survival data. The proportion of patients stopping trial medication during the one-year follow-up was estimated. *Results*. A total of 171 patients were included with a median prior duration of antisecretory treatment of four years (range: 0.5 to 14.6 years). 86 patients received esomeprazole 40 mg and 85 patients received placebo. At 12 months, statistically significantly more patients in the placebo group had discontinued (73% (62/85)) compared with the esomeprazole group (21% (18/86); *p* < 0.001). *Conclusions*. Long-term users of antisecretory drugs showed a preference for the active drug compared to placebo. However, 27% of patients continued on placebo throughout the study and did not need to reinstitute usual treatment. One in five patients treated with esomeprazole discontinued trial medication due to unsatisfactory symptom control. Discontinuation of antisecretory treatment should be considered in long-term users of antisecretory drugs. This trial is registered with Trial registration ClinicalTrials.gov ID: NCT00120315.

## 1. Introduction

The use of acid-suppressive medication is rapidly increasing. In the western world, proton pump inhibitors (PPIs) are second only to statins in expenditures, and antisecretory medication constitutes a substantial part of the medical budget in the Denmark and other western countries [1–3]. Most of the increased use of antisecretory medication is accounted for by long-term users [4, 5].

Antisecretory medication is highly effective for the treatment of peptic ulcer and reflux disease, but it has only sparse effect in the treatment of patients with functional dyspepsia [6]. In primary care, the most used strategy when investigating and treating dyspeptic patients is empirical treatment with antisecretory drugs [7]. As no data exist regarding the optimal treatment period, and as there is a poor correlation between initial treatment response and continued effect, some patients might have an unnecessary continued use of antisecretory medication [8]. In a Swedish study, Björnsson et al. [9] found that discontinuation of antisecretory mediation was successful in 27% of long-term users of PPI. Placebo-controlled discontinuation studies of antisecretory medication with interventions carried out in primary care are however not yet published.

The aim of the present study was therefore in a placebo-controlled randomised design to investigate if patients on long-term antisecretory medication need to continue treatment to control symptoms in a primary care setting.

## 2. Methods

### 2.1. Protocol

A total of 146 general practitioners in Denmark were visited, introduced to, and educated in study procedures and signed in as coinvestigators on the required forms from the Danish Medicines Agency (Protocol ID: 2612-2176). Eligible patients were evaluated, enrolled, and followed by their general practitioner from January 2003 to September 2006.

### 2.2. Assignment

Patients older than 17 years with a use of antisecretory treatment (proton pump inhibitors or histamine-2-receptor-antagonists) for at least 56 days during the previous six months were included. Exclusion criteria were alarm symptoms (gastrointestinal bleeding, iron deficiency anemia, progressive unintentional weight loss, progressive dysphagia, persistent vomiting, and epigastric mass on palpation), drug or alcohol abuse, serious or terminal diseases, planned hospitalization during follow-up, allergy to trial medication, and pregnant or fertile females not using safe contraception. Furthermore, patients suffering from oesophagitis, having had prior complicated peptic ulcer, and patients with prior endoscopically verified ulceration and ongoing use of nonsteroidal anti-inflammatory drugs (NSAIDs)/acetylic salicylic acid (ASA) were not eligible.

### 2.3. Interventions

Long-term users of antisecretory treatment were randomised to continued antisecretory treatment or placebo. Esomeprazole (Nexium, AstraZeneca) 40 mg or* identical* placebo was used as the randomised trial medication with a maximum of one tablet a day mimicking the patient's usual treatment. When trial medication was discontinued due to insufficient symptom control, reinstitution of usual antisecretory treatment was left to the GPs' discretion.

### 2.4. Masking

Both active medication and placebo were manufactured and packed by AstraZeneca, Mölndal, Sweden, in accordance with Good Manufactory Practice (GMP). Trial drug was delivered in identical, sealed containers holding 100 tablets. The local university hospital pharmacy (Fyns Amts Centralapotek, J. B. Winsløws Vej 13, DK-5000 Odense C, Denmark) labelled and randomised the trial drug accordingly using a computer-generated random number block randomisation with a fixed block size of four and a placebo/treatment ratio of one. The pharmacy kept the sequence of allocation concealed throughout the one-year follow-up, thus blinding patients, general practitioners, and researchers. Following seven days of discontinuation of usual antisecretory treatment with allowance of antacids as rescue treatment,* Helicobacter pylori* status was investigated with a ^13^C-urea breath test performed in general practice. During the seven days of discontinuation symptoms, demographics and clinical baseline characteristics of participants were obtained. Eradication therapy was given to any* Helicobacter-positive* patient (amoxicillin, clarithromycin, and esomeprazole, alternatively metronidazole if allergic to penicillin). The study was monitored according to Good Clinical Practice (GCP) by the GCP unit at Odense University Hospital, Denmark (Project number 02-004).

### 2.5. Outcomes

Primary endpoint was the time to discontinuation with trial medications (esomeprazole or placebo) due to the participants' need to change back to their usual antisecretory medication, that is, failure to control symptoms with the trial medication at any time point in the one-year follow-up. The proportion of patients stopping trial medication during the one-year follow-up was estimated. Additional analysis was carried out using health-related quality-of-life (MOS Short Form-36) [10] and generic dyspeptic quality-of-life (gastrointestinal symptom rating scale) [11] questionnaires.

### 2.6. Sample Size and Analysis

We assumed 15–20% of long-term users to be suffering from peptic ulcer disease, 40–45% to be suffering from nonerosive reflux disease, and approximately 40% to be suffering from functional dyspepsia [12]. Based on these diagnoses, we estimated the recurrence of symptoms in 10% in the esomeprazole group and 30% in the placebo group and given a type I error of 5% and a type II error of 20% at least 111 patients in each group were needed.

During data analysis, patients remained in the two arms of the study without revealing the intervention, thus allowing a blinded data analysis of the primary endpoint [13]. Primary outcome was assessed using survival data analysis. An intention to treat analysis was performed. Assumptions that dropouts in both groups had insufficient control of symptoms and therefore were analysed as having stopped with trial medication were made.

## 3. Results

### 3.1. Participant Flow and Follow-Up

**Figure 1:**
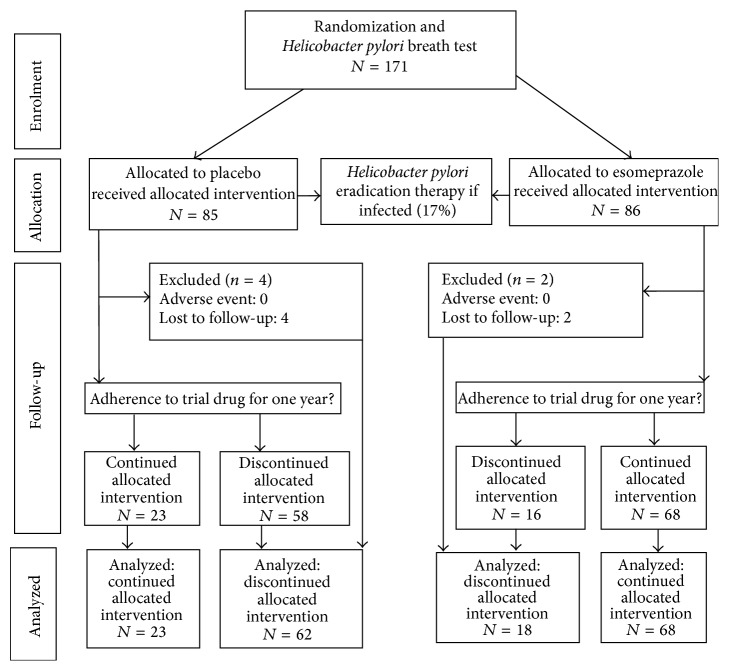
Flow of participants through study.

A total of 171 patients found eligible by their general practitioners were accepted to participate in the study (Figure 1); 17% were* Helicobacter pylori*-positive. During the one-year follow-up, six patients were lost to follow-up; no adverse events were seen, except for one patient developing a black discoloration of the tongue during* Helicobacter pylori* eradication treatment. Characteristics at baseline are shown in Table 1. No differences were found between GPs not including participants compared with active coinvestigators with regard to sex, single-handed or partnership practice, and number of long-term users, data not shown.

### 3.2. Analysis

**Figure 2:**
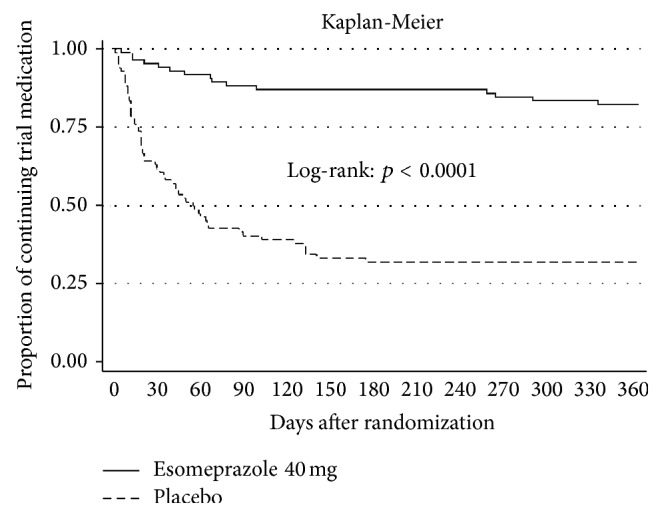
Discontinuation of trial medication in 1-year follow-up.

At the one-year follow-up, the stop of the trial medication had occurred more frequently (*p* < 0.0001, log-rank) in the placebo group compared with the group receiving esomeprazole. A total of 18/86 (21%) of participants treated with esomeprazole had stopped the trial medication compared to 62/85 (73%) of participants treated with placebo (Figure 2). Gastrointestinal symptom scores improved in both groups during the trial, but no statistically significant differences were found between the two groups after one year (Table 2). Quality-of-life scores were unchanged, and no differences were found between groups after one year (data not shown).

Table 3 shows a comparison between patients continuing placebo versus patients who stopped treatment with placebo. Statistically significantly more men stopped placebo treatment during follow-up (*p* = 0.003). A statistically nonsignificant tendency towards a more successful continuation with placebo treatment was found for patients with less gastrointestinal symptoms (total GSRS score, *p* = 0.07). The subscore regarding reflux symptoms (heartburn and regurgitation) did, however, show less influence on continued placebo treatment (*p* = 0.29).

## 4. Discussion

The present study provides estimates from primary care on the need for continued antisecretory treatment among long-term users. In a randomised, placebo-controlled trial, long-term users of antisecretory treatment were randomised to either continued antisecretory treatment (esomeprazole 40 mg) or placebo. We found that 27% of participants receiving placebo did not stop the trial medication during the complete follow-up.

To our knowledge, this study was the first nonindustry initiated drug trial conducted in a primary care setting, which was GCP monitored by a public GCP unit. The randomised design and blinding in the design hindered any contamination between participants in the same practice and allowed a blinded data analysis. The primary care setting is important as 90% of antisecretory drugs are prescribed in primary care [14] and the findings may be applicable to patients managed in primary care in similar settings.

Recruitment of patients for the study tended to be slower than expected and only 38% of the coinvestigators (GPs) recruited participants for the study. No differences were, however, found between GPs not including participants compared with active coinvestigators.

Based on register data [15], a large group of persons met the definition of long-term use of antisecretory treatment [4], but only a minority of the persons was included in this particular study. However, the register data do not provide information about indications for the prescription of antisecretory medication and some of the persons in the registers were probably meeting one or more of the exclusion criteria defined for the study. However, we cannot rule out the possibility that some of the persons, who were not included, had already tried to discontinue their antisecretory treatment.

Due to the absence of consensus on the optimal treatment duration, the design of a discontinuation study was chosen and the discontinuation of trial drug was the primary outcome measure. The fluctuation of dyspepsia symptoms is well known and severity as well as symptoms may vary in the same patient over time [16]. This may give rise to reflections on whether disappearance of symptoms due to the natural history of symptom fluctuation would allow participants receiving placebo to continue the trial drug. However, discontinuation of the trial drug largely took place during the first two months of follow-up and no discontinuation occurred during the last six months in the placebo group.

Rebound acid secretion following discontinuation may occur [17–20]. In this study, a potential rebound effect may have been an important factor and would result in an underestimation of the proportion of participants who succeeded in a continuation of placebo. Possible tapering of PPIs would probably be more appropriate than abrupt termination. However, the study also elucidated that if symptoms do not recur within the first 60 days of PPI withdrawal they are unlikely to recur. No endoscopy was performed prior to inclusion, which might be a limitation, as patients with uninvestigated dyspepsia on long-term treatment with PPI could in principle suffer from erosive esophagitis and therefore should probably not have withheld treatment.

This is the first placebo-controlled study on discontinuation of an acid-suppressive drug in a group of long-term users, with intervention carried out in primary care, but the results are in line with those of Björnsson et al. [9], who found that discontinuation was successful in 27% of long-term users. Their patients had symptom profiles very similar to those of our patients, but patients were allocated to taper down or constant dose before discontinuation and placebo was not introduced.

A number of studies examined discontinuation of PPI but without inclusion of a placebo group. A recent study by Murie et al. [21] included patients with a diagnosis of gastroesophageal reflux disease or functional dyspepsia for prospective intervention of patient education given at specialist nurse clinic. PPI was successfully discontinued in 34% and more than half of the patients reduced the PPI dose. In a study by Krol et al. [22], GPs were randomized to give usual care or sending a simple information leaflet about dyspepsia to patients treated with PPI for at least 12 weeks. A higher discontinuation rate (24%) was demonstrated in the intervention group, but the difference between groups did not persist after 20 weeks.

It may be objected that placebo treatment is not equivalent to absence of therapy and that 50–60% of patients with gastroesophageal reflux disease are satisfied with placebo on demand [23]. On the other hand, the perception of placebo is changing [24, 25] and in the present study symptoms were controlled for more than 6 months on placebo. Finally, psychological factors are of some importance in heartburn suffers [26]. Thus, on balance, it seems reasonable to consider stopping use of acid-suppressive drugs in a substantial number of long-term users without complicated peptic ulcer, severe oesophagitis, or need for protection against NSAIDs. Considerable savings could be expected if these results are applicable to the entire population of long-term users and future studies should concentrate on delineating factors predictive of successful discontinuation of proton pump inhibitors.

We recommend that symptom-based long-term antisecretory treatment in general practice should be interrupted by attempts at discontinuation. In our placebo-controlled study, nearly one-third of the patients were satisfied with placebo treatment. One in five patients treated with esomeprazole had even unsatisfactory symptom control and discontinued trial medication. Deleterious effects of antisecretory drugs have been reported [5, 27–30], and unnecessary treatment may lead to polypharmacy and unnecessary expenses for the patient and society. Randomised, controlled discontinuation trials, such as the study presented in this paper, are uncommon, but they may be useful in other areas of medicine (e.g., treatment with NSAIDs).

## Figures and Tables

**Table 1:** Demographics and clinical baseline characteristics of participants.

	Placebo	PPI^*∗*^
	*N* = 85	*N* = 86
Male (%)	37 (44)	41 (48)
Age, median (10%–90% percentile), year	59 (39–79)	59 (39–77)
Smoking, *n* (%; 95% CI)	31 (36; 26–48)	39 (45; 35–56)
Alcohol use, <21 units/weeks *n* (%; 95% CI)	79 (93; 85–97)	79 (92; 84–97)
Symptom duration, median (10%–90% percentile), year	4 (1–16)	4 (1–13)
*Helicobacter pylori-*positive *n* (%; 95% CI)	12 (14; 7–23)	17 (20; 12–30)
Previous endoscopy, *n* (%; 95% CI)	46 (54; 42–64)	39 (45; 34–56)
Previous NSAID/ASA use, *n* (%; 95% CI)	52 (61; 49–71)	53 (61; 50–72)

^*∗*^Esomeprazole 40 mg.

**Table 2:** Gastrointestinal symptoms at baseline and after 1 year according to randomization group.

Gastrointestinal symptom rating scale, median (IQR)^†^	Baseline		1 year		*p* value^‡^
	Placebo	PPI^*∗*^	Placebo	PPI^*∗*^	
	*N* = 85	*N* = 86	*N* = 69	*N* = 79	
Total score	2.6 (2.4; 2.8)	2.5 (2.3; 2.7)	1.9 (1.7; 2.1)	1.8 (1.7; 2.0)	0.73
Abdominal pain	2.7 (2.4; 3.0)	2.6 (2.4; 2.8)	2.0 (1.8; 2.3)	1.8 (1.6; 2.0)	0.58
Reflux	3.2 (2.8; 3.5)	3.1 (2.7; 3.4)	1.8 (1.6; 2.0)	1.8 (1.6; 2.0)	0.74
Indigestion	3.1 (2.8; 3.3)	3.0 (2.7; 3.2)	2.2 (2.0; 2.5)	2.2 (1.9; 2.4)	0.83
Diarrhea	2.2 (1.9; 2.5)	1.9 (1.6; 2.1)	1.6 (1.4; 1.8)	1.5 (1.3; 1.7)	0.48
Constipation	2.2 (1.9; 2.5)	2.0 (1.7; 2.3)	1.8 (1.5; 2.0)	1.7 (1.5; 2.0)	0.88

^*∗*^Esomeprazole 40 mg.

^†^Gastrointestinal symptom rating scale. Range 1–7. Increasing values reflect increasing symptoms.

^‡^Between group comparison. Difference in GSRS score at entry versus one year. Conditional linear regression adjusted for GP clusters.

**Table 3:** Associations with discontinuation in placebo group (*n* = 85).

	Successful discontinuation (continuing placebo) (*n* = 23)	Unsuccessful discontinuation (discontinuing placebo) (*n* = 62)	*p* value^*∗*^
Age median (IQR), year	58 (48–62)	60 (51–69)	0.81
Male (%)	23%	58%	0.003
*Helicobacter pylori-*positive, % (95% CI)	29 (12–51)	17 (8–29)	0.24
No nicotine use, % (95% CI)	68 (46–85)	50 (37–63)	0.12
Alcohol use, <21 units/weeks, % (95% CI)	92 (74–99)	92 (82–97)	0.96
Symptom duration, median (10%; 90%) percentile, year	4 (0–13)	4 (1–14)	0.21
Previous endoscopy, % (95% CI)	40 (21–61)	47 (24–60)	0.58
Previous NSAID/ASA use, % (95% CI)	60 (39–79)	62 (48–74)	0.89
GSRS total, mean (95% CI)	2.5 (2.2–2.8)	2.9 (2.5–3.2)	0.07
GSRS reflux score, mean (95% CI)	3.0 (2.5–3.5)	3.3 (2.9–3.8)	0.29
SF-36 physical health summary score, mean score (95% CI)	41 (36–46)	43 (40–46)	0.54
SF-36 mental health summary score, mean score (95% CI)	48 (42–54)	51 (49–54)	0.20

^*∗*^ *F*-test for linear regression.
